# Impact of interhemispheric inhibition on bimanual movement control in young and old

**DOI:** 10.1007/s00221-021-06258-7

**Published:** 2022-01-12

**Authors:** Takuya Morishita, Jan E. Timmermann, Robert Schulz, Friedhelm C. Hummel

**Affiliations:** 1 Defitech Chair of Clinical Neuroengineering, Center for Neuroprosthetics and Brain Mind Institute, Swiss Federal Institute of Technology (EPFL), Campus Biotech, Chemin des Mines, 9, 1202 Geneva, Switzerland; 2 Defitech Chair of Clinical Neuroengineering, Center for Neuroprosthetics and Brain Mind Institute, Swiss Federal Institute of Technology Valais (EPFL Valais), Sion, Switzerland; 3 Department of Neurology, University Medical Center Hamburg-Eppendorf, Martinistr. 52, 20251, Hamburg, Germany; 4 Clinical Neuroscience, University of Geneva Medical School, Geneva, Switzerland

**Keywords:** TMS, Paired-pulse TMS, Interhemispheric inhibition, Bimanual movement, Aging

## Abstract

Interhemispheric interactions demonstrate a crucial role for directing bimanual movement control. In humans, a well-established paired-pulse transcranial magnetic stimulation paradigm enables to assess these interactions by means of interhemispheric inhibition (IHI). Previous studies have examined changes in IHI from the active to the resting primary motor cortex during unilateral muscle contractions; however, behavioral relevance of such changes is still inconclusive. In the present study, we evaluated two bimanual tasks, i.e., mirror activity and bimanual anti-phase tapping, to examine behavioral relevance of IHI for bimanual movement control within this behavioral framework. Two age groups (young and older) were evaluated as bimanual movement control demonstrates evident behavioral decline in older adults. Two types of IHI with differential underlying mechanisms were measured; IHI was tested at rest and during a motor task from the active to the resting primary motor cortex. Results demonstrate an association between behavior and short-latency IHI in the young group: larger short-latency IHI correlated with better bimanual movement control (i.e., less mirror activity and better bimanual anti-phase tapping). These results support the view that short-latency IHI represents a neurophysiological marker for the ability to suppress activity of the contralateral side, likely contributing to efficient bimanual movement control. This association was not observed in the older group, suggesting age-related functional changes of IHI. To determine underlying mechanisms of impaired bimanual movement control due to neurological disorders, it is crucial to have an in-depth understanding of age-related mechanisms to disentangle disorder-related mechanisms of impaired bimanual movement control from age-related ones.

## Introduction

Bimanual movement control is an important form of control of dexterous movements for crucial daily life activities; loss of bimanual movement control leads to relevant functional impairment of dexterity impacting on daily living. To direct complex bimanual movements, distinct cortical and subcortical areas are involved in production and control of bimanual movement. It has been demonstrated that they rely on an orchestrated interplay of distinct interactions of bihemispheric cortical areas via the corpus callosum (for review, Gooijers and Swinnen 2014).

In humans, such interhemispheric interactions can be investigated in-vivo, non-invasively by well-established dual-site paired-pulse transcranial magnetic stimulation (TMS) paradigms (Ferbert et al. 1992). Specifically, interhemispheric inhibition (IHI) can be assessed by analyzing the effect of a conditioning stimulus (CS) to one primary motor cortex on the size of motor-evoked potential (MEP) amplitude evoked by the application of a test stimulus (TS) to the opposite primary motor cortex at interstimulus intervals (ISIs) between 6 and 50 ms. As IHI was reduced in patients with callosal infarction (Li et al. 2013), IHI has been considered to be predominantly mediated via the corpus callosum. Two types of IHI are known (Ni et al. 2009)—short-latency IHI (S-IHI) and long-latency IHI (L-IHI) corresponding to different underlying mechanisms (different gamma-aminobutyric acid (GABA) receptors: Irlbacher et al. 2007) and anatomical connections (callosal fibers: Li et al. 2013). It has been considered and discussed that IHI between the primary motor cortices may play an important role in suppressing activity of the contralateral side (mirror activity) contributing critically to bimanual movement control (e.g., Duque et al. 2007). Based on this view, the previous studies have investigated the effects of unilateral muscle contractions on IHI from the active to the resting primary motor cortex in a detailed neurophysiological manner (e.g., Ferbert et al. 1992; Perez and Cohen 2008). However, the results of previous studies are incoherent. S-IHI was increased during unilateral muscle contractions compared with the resting state (Ferbert et al. 1992; Perez and Cohen 2008; Vercauteren et al. 2008; Hinder et al. 2010; Morishita et al. 2012; Uehara et al. 2014); however, other studies reported decreased S-IHI (Perez and Cohen 2008; Nelson et al. 2009; Howatson et al. 2011; Sattler et al. 2012; Turco et al. 2019). In case of L-IHI, no modulation (Sattler et al. 2012; Morishita et al. 2014; Uehara et al. 2014; Turco et al. 2019) and decreased L-IHI (Nelson et al. 2009) were reported.

Although inconsistent findings could be attributable to methodological differences among previous studies to some extent, a missing aspect has not been considered within this framework: to interpret results, the behavioral relevance of IHI from the active to the resting primary motor cortex has to be taken into account. The degree of IHI itself may be considered as a function; however, TMS probes only a subgroup of descending motor fibers and by TMS descending connections contributing to voluntary movement are perhaps not equally activated and functionally relevant (for review, Bestmann and Krakauer 2015). Thus, an investigation of the behavioral relevance will extend the understanding of IHI by means of paired-pulse TMS during a unilateral muscle contraction.

Based on the previous findings briefly described above, we hypothesized that the degree of IHI from the active to the resting primary motor cortex during a unilateral muscle contraction would exhibit behavioral impact on the performance of bimanual movement control. For bimanual movement control, the previous studies have demonstrated evident decline in older adults (e.g., Swinnen 1998). Thus, aging is a valuable model to investigate the functional role of IHI for bimanual movement control. In line with this, the previous studies have also examined motor inhibition and its decline in older adults (for review, Levin et al. 2014); the methodology to study motor inhibition varies among studies such as different inhibitory circuits assessed by TMS (Fujiyama et al. 2012; Heise et al. 2013; Opie et al. 2015), concurrent TMS-electroencephalography (EEG) (Opie et al. 2018; Casula et al. 2020), and magnetic resonance imaging techniques (Hermans et al. 2018; Cuypers et al. 2020). To this date, with regard to age, only a few studies have examined IHI from the active to the resting primary motor cortex (Talelli et al. 2008; Hinder et al. 2010; Mooney et al. 2018; Ermer et al. 2020) using paired-pulse TMS during unilateral muscle contractions and the results are inconclusive in terms of bimanual movement control. In case of older adults, we tested the hypotheses if less IHI from the active to the resting primary motor cortex compared with young adults would be associated with poorer bimanual performance. We aimed to extend the neurophysiological understanding of transcallosal inhibition and its behavioral relevance, together with the aging aspect previously demonstrating a relevant behavioral change in bimanual movement control.

## Methods

### Participants

In total, 37 participants took part in the study: 15 young (8 females; mean age, 26.1 standard deviation: SD ± 3.3) years and 22 older (11 females; mean age, 65.0 ± 8.8) years). All participants were right-handed as assessed with the Edinburgh handedness inventory (mean score: young, 90.0 ± 13.6; older, 93.6 ± 8.5) (Oldfield 1971). They were naïve to the experimental purpose of the study, and none of them played a musical instrument regularly. None of the participants had a history of serious medical, neurological, psychiatric illnesses, or any contraindications for TMS, as probed by a standardized questionnaire (Rossi et al. 2009).

### Experimental protocol

The investigations consisted of TMS and behavioral experiments. To assess IHI between primary motor cortices, a well-established dual-site paired-pulse TMS paradigm (Ferbert et al. 1992; Chen et al. 2003; Ni et al. 2009; Liuzzi et al. 2010) was used in the TMS experiment. IHI was measured at rest and during weak isometric abduction of the right index finger. For the behavioral experiment, the participants performed two bimanual tasks to assess involuntary mirror activity and performance of bimanual anti-phase tapping (details below). These were chosen based on the previous findings showing behavioral differences between young and older adults (mirror activity: Cincotta et al. 2006; Hinder et al. 2011, 2013) (bimanual asymmetric movement: Fling and Seidler 2012; Serbruyns et al. 2015; Fujiyama et al. 2016a). Both tasks represent, though probably to a different extent, the individual ability to control both hands independently. The behavioral experiment was performed after completion of the TMS experiment to minimize biases of the investigators for the TMS recording from the behavioral experiment, as especially individual performance of bimanual anti-phase tapping was apparent.

### EMG recording

Pairs of Ag/AgCl electrodes were used for surface EMG recordings taken from the right and left first dorsal interosseous muscles (FDI). The active electrode was placed over the muscle belly and the reference electrode over the metacarpo-phalangeal joint of the index finger. The EMG signal was amplified (× 1000) and filtered (bandwidth, 10–2 kHz, CED 1902 amplifier, Cambridge Electronic Design, Cambridge, UK). All signals were digitized at a sampling rate of 5 kHz and stored on a computer for offline analysis (Signal software version 4.05, Cambridge Electronic Design, Cambridge, UK). EMG recordings were used for the assessments of IHI by the paired-pulse TMS paradigm and mirror activity.

### TMS and assessment of IHI

**Fig. 1:**
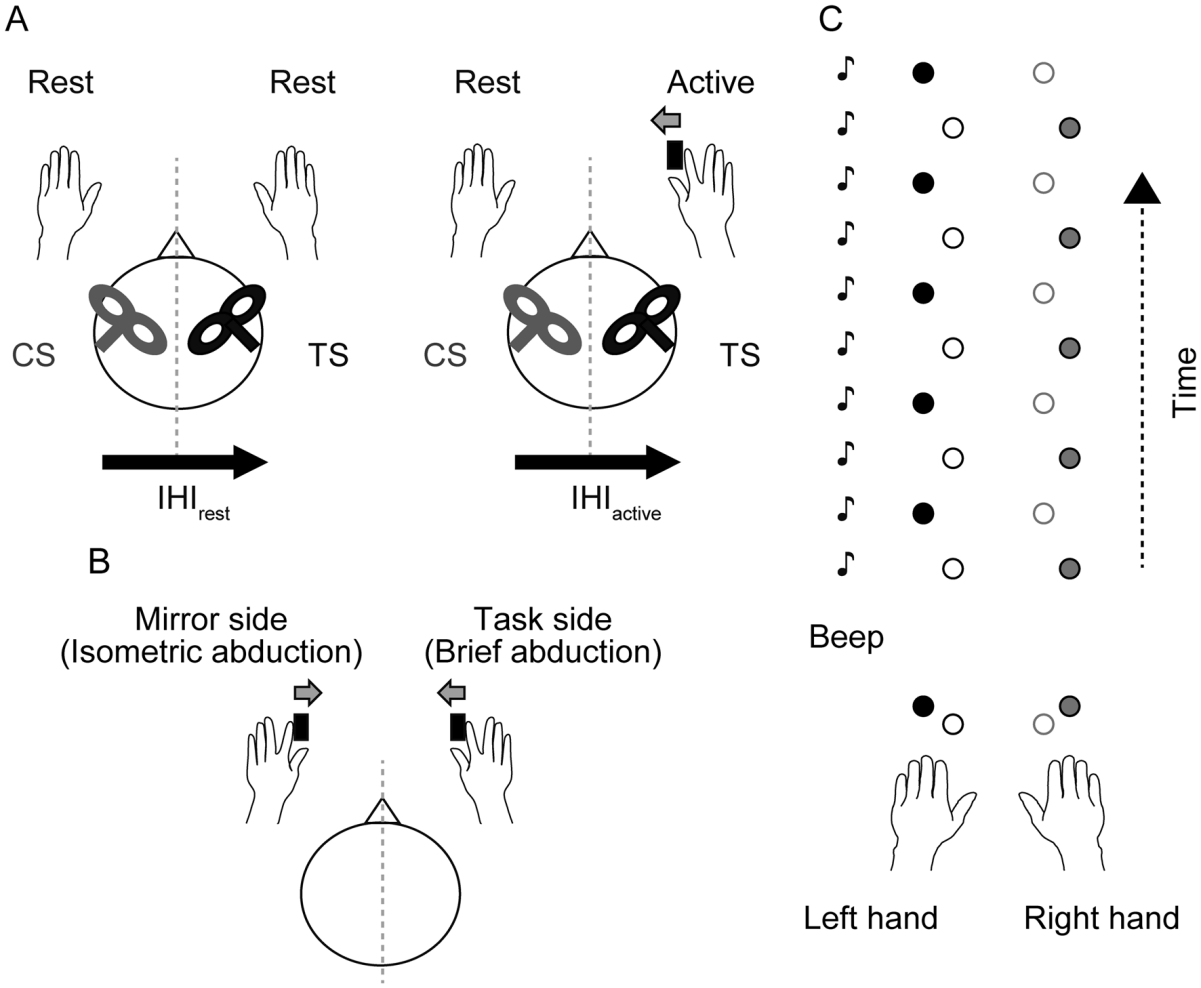
Experimental setup. **A** Interhemispheric inhibition (IHI). A well-established dual-site paired-pulse transcranial magnetic stimulation (TMS) paradigm (Ferbert et al. 1992) was used for assessing IHI as transcallosal inhibition. The conditioning stimulus (CS) was delivered to the left primary motor cortex and the test stimulus (TS) was delivered to the right primary motor cortex. Two states of IHI were measured: IHI at rest (left, IHI_rest_) and IHI during weak isometric abduction of the right index finger (right, IHI_active_) (Talelli et al. 2008; Hinder et al. 2010). **B** Mirror activity. A modified method of Mayston et al. (1999) and Hübers et al. (2008) was used. With their index fingers, the participants were instructed to perform brief abduction in the task hand while maintaining weak isometric abduction (~ 15% of individual maximum voluntary contraction) in the mirror hand. **C** Bimanual anti-phase tapping. Participants were instructed to perform a bimanual rhythmic finger-tapping task using their index and middle fingers as described previously (Liuzzi et al. 2011). We looked for the maximum frequency at which the anti-phase tapping could be maintained for 20 s

Two branding-iron figure-of-eight coils (50 mm in diameter) were separately connected to two Magstim 200^2^ stimulators (The Magstim Company, Whitland, UK). The principle of the dual-site paired-pulse TMS paradigm for assessing IHI is based on analyzing the effect of a CS to one primary motor cortex on the size of MEP amplitude evoked by the application of a TS to the opposite primary motor cortex. First, the participants performed 2–3 maximum isometric abduction of the right index finger for ~ 4 s. The maximum force was used to set a target for subsequent weak isometric abduction of the right index finger (~ 15% of individual maximum voluntary contraction) (Talelli et al. 2008; Hinder et al. 2010). Force exerted with the index finger was measured by a load cell (model LMB-A-500N, KYOWA Electronic, Tokyo, Japan). The force signal was amplified and displayed on an oscilloscope placed in front of the participant for visual feedback. The optimal location to evoke MEPs (hotspot) and the resting motor threshold (RMT) for each FDI was determined. The coil was positioned tangentially over the scalp with an orientation inducing a posterior–anterior current perpendicular to the presumed central sulcus at ~ 45 degrees relative to the midsagittal line. RMT was defined as the minimum stimulator output that evoked peak-to-peak MEP amplitude of > 50 µV in at least five of ten consecutive trials (Rossini et al. 1994). The CS was delivered to the left primary motor cortex and the TS was delivered to the right primary motor cortex to measure IHI from the left to the right primary motor cortex. This direction was chosen based on the previous studies examining IHI from the active to the resting primary cortex in older adults (Talelli et al. 2008; Hinder et al. 2010; Mooney et al. 2018; Ermer et al. 2020). Two ISIs were adopted to assess IHI: 10 ms for S-IHI and 40 ms for L-IHI (Chen et al. 2003; Ni et al. 2009). In the present study, we measured IHI in two states: IHI at rest (i.e., condition IHI_rest_, Fig. 1A left) and IHI during weak isometric abduction of the right index finger (i.e., condition IHI_active_, Fig. 1A right). For IHI_active_, the participants were instructed to maintain the predetermined weak isometric abduction of the right index finger (Talelli et al. 2008; Hinder et al. 2010). The TS intensity was adjusted to evoke peak-to-peak amplitude ~ 1 mV for each condition (i.e., IHI_rest_ and IHI_active_), and the CS intensity was kept at 130% RMT during the whole experiment (Hinder et al. 2010; Turco et al. 2019). For each condition, we performed three states, randomly varied at intervals of 5–8 s: 18 TS alone, 18 CS + TS with ISI 10 ms, and 18 CS + TS with ISI 40 ms (total of 2 conditions × 3 states × 18 trials = 108 trials).

### Assessment of bimanual movement control: mirror activity

Mirror activity can be defined as involuntary activity related to the timing of contralateral voluntary activity, considering an analogy with mirror movements in patients (Mayston et al. 1997). Our aim was to quantify the amount of mirror activity rather than the manifestation of mirror activity (e.g., Duque and Ivry 2009); thus, we modified a method used by Mayston et al. (1999) and Hübers et al. (2008) to quantify the amount of mirror activity in young participants (*n* = 15) and a subset of older participants (*n* = 15). A schematic illustration for assessing mirror activity is shown in Fig. 1B. First, the participants performed for 2–3 times a maximum isometric abduction of the left index finger for a duration of ~ 4 s. The maximum force was used to set a target for subsequent weak isometric abduction of the left index finger defined as ~ 15% of individual maximum voluntary contraction. The weak isometric abduction was maintained (mirror side), while the participants were instructed to perform brief abductions with their right index finger (task side). The participants were instructed to loosely follow the pace of an external trigger (frequency ~ 0.2 Hz) and performed brief abductions; we did not adopt a reaction time task to assess mirror activity. Force feedback was provided on the oscilloscope for the left side to maintain isometric abduction. Fifty trials were obtained (Hübers et al. 2008; Ohtsuka et al. 2012), and for the following offline analysis, we used a custom-written MATLAB script (version R2019a, The MathWorks, Natick, MA, USA): (1) According to the EMG onset of the right FDI, which was defined at 0.1 mV, waveforms were segmented from 400 ms before to 400 ms after the EMG onset; (2) each waveform was single-trial rectified and averaged; (3) in the averaged waveform, peak mirror activity in the left FDI was identified with a 25 ms time window; (4) then, mirror activity was calculated with the following formula:

$${\text{Mirror activity (}}\% {\text{ facilitation)}} = ({\text{Peak mirror activity}} - {\text{baseline}})/{\text{baseline}} \times {100}{\text{.}}$$

Peak mirror activity was defined as the maximum value of EMG area after baseline; baseline was determined as EMG area 400–200 ms before the onset of the right FDI EMG activity to obtain baseline not contaminated by increased pre-onset EMG activity (please see Results). Ohtsuka et al. (2012) also reported that EMG activity in the mirror side in healthy young adults increased before the EMG onset of the task side. Since the onset of mirror activity in healthy adults (Mayston et al. 1999)—as well as mirror movements in patients (Regli et al. 1967; Mayston et al. 1997)—was shown to be individual-dependent, we identified mirror activity by an individual peak (Hübers et al. 2008). As activity of the task side could influence the amount of mirror activity, the peak EMG amplitude of the right FDI (Peak Amp, in % of maximum voluntary contraction), peak time (Peak Time, in ms from the EMG onset of right FDI) (Hübers et al. 2008), and activity duration (in ms) for each participant were measured. Activity duration of the right FDI was defined as the time between the EMG onset of the right FDI and amplitude descent under 0.1 mV.

### Assessment of bimanual movement control: anti-phase tapping

The participants were instructed to perform a bimanual rhythmic finger-tapping task using their index and middle fingers as described previously (Liuzzi et al. 2011). Custom-made keypads connected to a personal computer were used to record the finger taps. The participants were instructed to perform discrete anti-phase tapping: synchronous tapping of the left index/right middle finger alternating with synchronous tapping of the left middle/right index finger (Fig. 1B). The tapping frequency was auditory paced by the computer, while visual cues (start and stop signal, and fixation cross) were provided on a personal computer screen. Presentation software (version 0.61, Neurobehavioral System, Berkeley, USA) was used for the experimental setup and to record the timing of the key presses. The participants listened to the auditory beep sound for 10 s to catch the paced rhythm, while looking at a fixation cross. Then they were instructed to join the rhythm after a “GO” signal by maintaining the anti-phase tapping for 20 s in the same frequency. The participants were asked to tap in tune with the auditory beep sound. We looked for the maximum frequency at which anti-phase tapping could be maintained for 20 s in two out of three trials, and we increased the frequency in 20 beats per minute (bpm) steps. The performance was analyzed offline using a custom-written MATLAB script (version R2019a, The MathWorks, Natick, MA, USA), and the following two events were considered as errors: (1) occurrence of a phase transition; (2) tapping stop. One familiarization block consisting of three trials at 60 bpm was provided prior and the measurement began at 60 bpm. If difficulty of performing anti-phase tapping was seen during the initial instruction, 40 bpm was used for familiarization and starting frequency.

### Data and statistical analysis

Peak-to-peak MEP amplitude for each trial was measured offline. IHI was expressed as a percentage of the mean test MEP amplitude evoked by the TS alone ([CS + TS]/TS alone × 100). Values below 100% indicate inhibition and values above 100% indicate facilitation. We carefully checked EMG activity in the left FDI; IHI could be contaminated by changes of EMG activity in the left FDI (Chen et al. 2003). Trials with EMG activity exceeding > 25 µV from baseline (100 ms prior to the TS) were excluded from the subsequent analysis (Muellbacher et al. 2000). In total, 93.7% of trials were used for the subsequent analyses (IHI_rest_ young, 98.3%; IHI_rest_ older, 92.1%; IHI_active_ young, 98.6%; IHI_active_ older, 85.9%). Three older participants exhibited evident EMG activity in the left FDI during weak isometric abduction of the right FDI (i.e., IHI_active_), which might be considered as mirror activity. Trial exclusion resulted in no reliable trials in IHI_active_ in these participants. The MEP analysis was not conducted blindly considering two bimanual tasks; however, the analysis was conducted objectively following the criteria mentioned above.

We hypothesized that IHI values would reveal differences between the two groups. For this, repeated-measures ANOVA was used: (1) to evaluate the effects of STATE (2 levels: rest, active) as a within participants factor and AGE (2 levels: young, older) as a between participants factor on the test MEP amplitude, and; (2) to analyze the effects of ISI (2 levels: 10 ms, 40 ms) and STATE (2 levels: rest, active) as within participants factors and the factor AGE (2 levels: young, older) as a between participants factor on IHI. However, there was no difference of IHI between the two groups (please see “Results”) and we noticed that IHI values in older adults despite the factor STATE were similar; thus, we explored whether relationships of IHI between the resting and active state were similar between the two groups. We estimated individual linear models with IHI_active_ as an outcome (dependent variable), using the function lm in R for Statistical Computing, including AGE, IHI_rest_, and AGE × IHI_rest_ interaction terms. S-IHI and L-IHI were modeled separately.

One young participant had missing behavioral data due to an incomplete recording. Five participants in the older group were unable to maintain anti-phase tapping for 20 s even at 40 bpm. Two-tailed unpaired *t* test (Welch’s *t* test) was used to compare the amount of mirror activity and the performance of anti-tapping task between the two groups. Additionally, it was used to compare activity of the task side for the assessment of mirror activity (i.e., Peak Amp, Peak Time, and activity duration). To test our hypothesis whether IHI contributes to the amount of mirror activity and the performance of anti-tapping task, we estimated individual linear models including AGE, IHI, and AGE × IHI interaction terms. Each IHI value (i.e., S-IHI_rest_, S-IHI_active_, L-IHI_rest_, L-IHI_active_) was modeled separately. For each model, the interaction was excluded from the model to see a main effect of IHI in case the interaction was not significant.

Normal distribution was tested using the Shapiro–Wilk test before all statistical parametric testing was applied. The level of significance was set at *p* < 0.05 for all tests. Partial eta-squared values are presented as measures of effect size. R for Statistical Computing (version 4.0.5 for Windows) was used for all statistical analyses. Values are presented with SD.

## Results

### IHI

TMS parameters for each group are summarized in Table 1. Repeated-measures ANOVA for the test MEP amplitude revealed no significant effects of STATE (*F*_(1, 32)_ = 0.469, *p* = 0.499, *η*^2^_*p*_ = 0.014), AGE (*F*_(1, 32)_ = 0.002, *p* = 0.965, *η*^2^_*p*_ < 0.001), or their interaction (*F*_(1, 32)_ = 1.470, *p* = 0.234, *η*^2^_*p*_ = 0.044), indicating that the test MEP amplitude was well adjusted.

**Table 1:** Summary of TMS parameters in young and older groups

Group	RMT (% of MSO)		1 mV intensity (% of RMT)		Test MEP amplitude (mV)	
	Left	Right	Rest	Active	IHI_rest_	IHI_active_
Young	37.8 (8.4)	38.1 (8.7)	124.0 (15.3)	118.8 (16.0)	1.00 (0.42)	1.16 (0.50)
Older	38.1 (5.7)	38.9 (6.3)	127.4 (12.8)	122.4 (11.5)	1.10 (0.53)	1.05 (0.42)

Test motor-evoked potential (MEP) amplitude was adjusted to ~ 1 mV in each condition (i.e., IHI_rest_ and IHI_active_, Fig. 1A)

*IHI* interhemispheric inhibition; *MSO* maximum stimulator output; *RMT* resting motor threshold

Repeated-measures ANOVA for IHI revealed significant main effects of factor ISI (*F*_(1, 32)_ = 8.034, *p* = 0.008, *η*^2^_*p*_ = 0.201), STATE (*F*_(1, 32)_ = 7.985, *p* = 0.008, *η*^2^_*p*_ = 0.200), but not AGE (*F*_(1, 32)_ = 0.681, *p* = 0.415, *η*^2^_*p*_ = 0.021). There was also no significant ISI × STATE interaction (*F*_(1, 32)_ = 0.057, *p* = 0.812, *η*^2^_*p*_ = 0.002). Importantly, there were no significant AGE interactions (ISI × AGE, *F*_(1, 32)_ = 0.915, *p* = 0.346, *η*^2^_*p*_ = 0.028; STATE × AGE, *F*_(1, 32)_ = 0.362, *p* = 0.552, *η*^2^_*p*_ = 0.011; ISI × STATE × AGE, *F*_(1, 32)_ = 0.024, *p* = 0.877, *η*^2^_*p*_ = 0.001), indicating that the IHI values were similar on a group level between the two groups irrespective of the factors ISI and STATE.

**Fig. 2:**
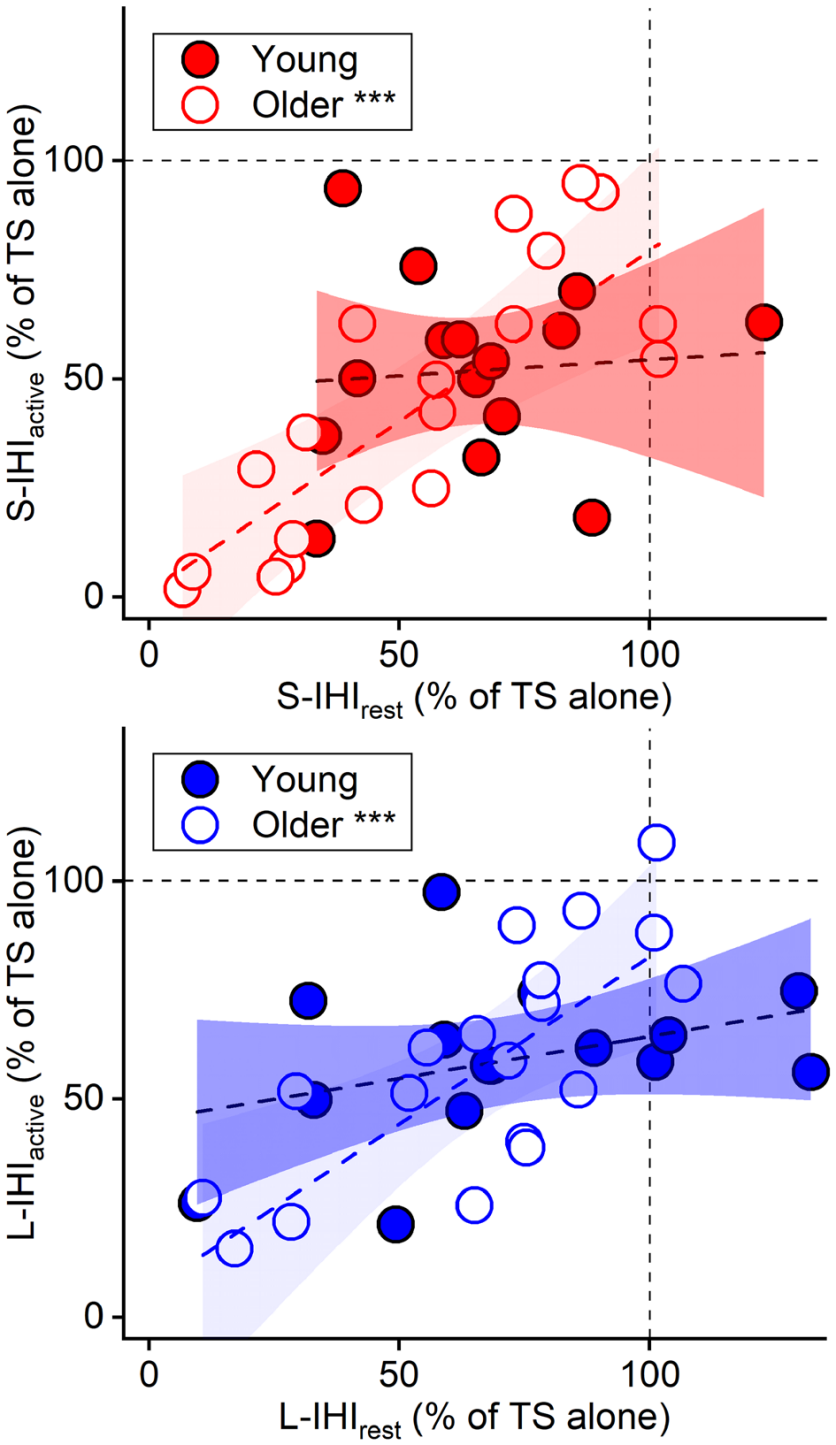
IHI_active_ and IHI_rest_ (with 95% confidence interval). The analysis showed a significant AGE × S-IHI_rest_ interaction (*p* = 0.008, *η*^2^_*p*_ = 0.215, Table 2), indicating that S-IHI_rest_ predicts the degree of S-IHI_active_ (dependent variable) differently in the young and older groups: a significant main effect of S-IHI_rest_ in the older group (*p* < 0.001, *η*^2^_*p*_ = 0.502) but not in the young group (*p* = 0.747, *η*^2^_*p*_ = 0.004). *Bottom*: The analysis also demonstrated a significant AGE × L-IHI_rest_ interaction (*p* = 0.017, *η*^2^_*p*_ = 0.174, Table 2): a significant main effect of L-IHI_rest_ in the older group (*p* < 0.001, *η*^2^_*p*_ = 0.419) but not in the young group (*p* = 0.175, *η*^2^_*p*_ = 0.060) (dependent variable: L-IHI_active_). The *y*-axis indicates the amplitude of the conditioned MEP amplitude during a unimanual contraction (top, S-IHI_active_; bottom, L-IHI_active_) and the *x*-axis indicates the amplitude of the conditioned MEP amplitude at rest (top, S-IHI_rest_; bottom, L-IHI_rest_); the conditioned MEP amplitude is expressed as a percentage of the mean test MEP amplitude evoked by the TS alone showing the degree of IHI ([CS + TS]/TS alone × 100). The vertical and horizontal dashed lines indicate the MEP amplitude evoked by the TS alone (100%). Values below 100% indicate inhibition and values above 100% indicate facilitation. The statistical analysis was conducted with linear model including AGE, IHI_rest_, and AGE × IHI_rest_ interaction terms, and then separately in the young and older groups since the AGE × IHI_rest_ interactions were significant (Table 2). *N* = 15 in young and *N* = 19 due to missing data (3 older without IHI_active_, please see the main text). ***p < 0.001

Contrary to our hypothesis, the IHI values between the two groups were similar. We explored relationships of IHI between the resting and active state using linear model analysis (Fig. 2 and Table 2). The analysis revealed significant AGE × IHI_rest_ interactions (S-IHI_active_, *p* = 0.008, *η*^2^_*p*_ = 0.215; L-IHI_active_, *p* = 0.017, *η*^2^_*p*_ = 0.174, Fig. 2 and Table 2) with positive associations of IHI_rest_ and IHI_active_ in the older group (S-IHI_active_, *p* < 0.001, *η*^2^_*p*_ = 0.502; L-IHI_active_, *p* < 0.001, *η*^2^_*p*_ = 0.419) but not in the young group (S-IHI_active_, *p* = 0.747, *η*^2^_p_ = 0.004; L-IHI_active_, *p* = 0.175, *η*^2^_*p*_ = 0.060). The results indicate that the degree of IHI_active_ in older participants could be predicted by the degree of IHI_rest_.

**Table 2:** Linear model results of IHI_active_

Outcome	Predictor	Estimate	95% Confidence Interval		*p* value	*η*^2^_*p*_
			Lower	Upper		
S-IHI_active_	Intercept	− 1.27	− 20.48	17.94	0.894	
	AGE	48.37	11.60	85.13	0.012	0.194
	S-IHI_rest_	0.85	0.53	1.17	< 0.001	0.502
	AGE × S-IHI_rest_	− 0.78	− 1.33	− 0.22	0.008	0.215
L-IHI_active_	Intercept	11.66	− 10.68	34.01	0.295	
	AGE	33.46	1.86	65.06	0.039	0.135
	L-IHI_rest_	0.71	0.40	1.02	< 0.001	0.419
	AGE × L-IHI_rest_	− 0.52	− 0.94	-0.10	0.017	0.174

Linear model analysis was conducted with the older group as the reference category. *η*^2^_*p*_, partial eta-squared

### Mirror activity

The amount of mirror activity between the two groups demonstrated a significant difference (two-tailed unpaired *t* test, young vs. older, Mirror activity: 20.1 ± 8.1 vs. 27.9 ± 10.1, *t* = 2.322, df = 26.4, *p* = 0.028). Additionally, activity of the task side did not differ between the two groups (two-tailed unpaired *t* test, young vs. older, Peak Amp: 19.5 ± 7.3 vs. 20.7 ± 5.3, *t* = 0.520, d*f* = 23.7, *p* = 0.608; Activity duration: 186.7 ± 62.8 vs. 173.5 ± 74.1, *t* = − 0.519, d*f* = 26.8, *p* = 0.608) (Wilcoxon sum exact test, young vs. older, Peak Time: 75.2 ± 37.6 vs. 51.8 ± 46.1, *W* = 69, *p* = 0.123). Wilcoxon sum exact test was used for Peak Time, since the Shapiro–Wilk test in the older data showed a significant deviation from normality (*W* = 0.872, *p* = 0.037). As shown in a previous study (Ohtsuka et al. 2012), several participants showed peak mirror activity including the EMG area before the EMG onset of the right FDI (5 young and 6 older).

**Fig. 3:**
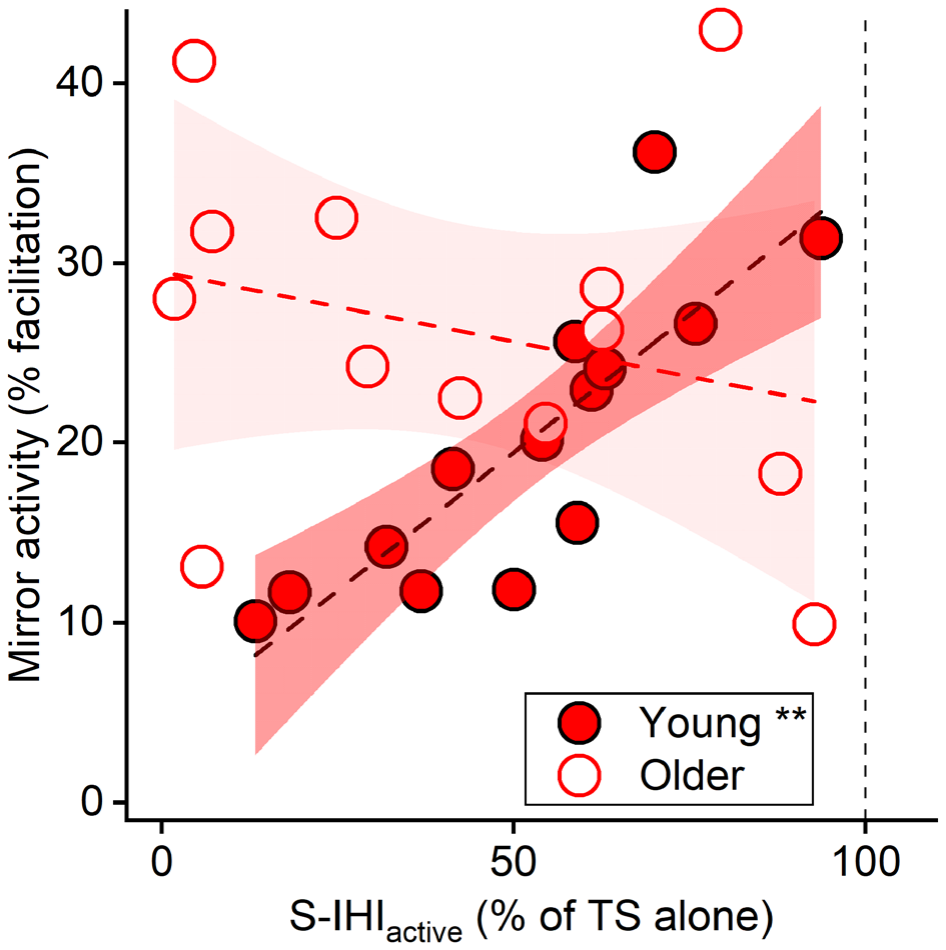
Mirror activity and S-IHI_active_ (with 95% confidence interval). The analysis showed a significant AGE × S-IHI_active_ interaction (*p* = 0.003, *η*^2^_*p*_ = 0.325, Table 3), indicating that S-IHI_active_ influences the amount of mirror activity (dependent variable) differently in the young and older groups: a significant main effect of S-IHI_active_ in the young group (*p* = 0.004, *η*^2^_*p*_ = 0.313) but not in the older group (*p* = 0.251, *η*^2^_*p*_ = 0.057). The *y*-axis indicates the amount of mirror activity (% facilitation) and the *x*-axis indicates the amplitude of the conditioned MEP amplitude expressed as a percentage of the mean test MEP amplitude evoked by the TS alone showing the degree of S-IHI_active_ ([CS) + TS]/TS alone × 100). The vertical dashed line indicates the MEP amplitude evoked by the TS alone (100%). Values below 100% indicate inhibition and values above 100% indicate facilitation. The statistical analysis was conducted with linear model including AGE, S-IHI_active_, and AGE × S-IHI_active_ interaction terms, and then separately in the young and older groups, since the AGE × S-IHI_active_ interaction was significant (Table 3). *N* = 14 in young and *N* = 13 due to missing data (1 young due to an incomplete recording; 2 older without S-IHI_active_, please see the main text). **p < 0.01

Linear model analysis revealed a significant AGE × S-IHI_active_ interaction (*p* = 0.003, *η*^2^_*p*_ = 0.325, Fig. 3 and Table 3) with a positive association of S-IHI_active_ and the amount of mirror activity in the young group (*p* = 0.004, *η*^2^_*p*_ = 0.313) but not in the older group (*p* = 0.251, *η*^2^_*p*_ = 0.057). In other words, the weaker short-latency inhibitory interaction from the active to the resting primary motor cortex (i.e., S-IHI_active_), the larger amount of mirror activity young participants exhibited. S-IHI_rest_ and L-IHI (i.e., L-IHI_rest_ and L-IHI_active_) did neither reveal significant interactions with AGE (Table 3), nor did they show significant contributions to the amount of mirror activity after excluding their interaction terms (S-IHI_rest_: *p* = 0.946, *η*^2^_*p*_ < 0.001; L-IHI_rest_: *p* = 0.412, *η*^2^_*p*_ = 0.026; L-IHI_active_: *p* = 0.610, *η*^2^_*p*_ = 0.011).

**Table 3:** Linear model results of mirror activity

Outcome	Predictor	Estimate	95% confidence interval		*p* value	*η*^2^_*p*_
			Lower	Upper		
Mirror activity	Intercept	30.38	20.64	40.12	< 0.001	
	AGE	− 16.57	− 34.31	1.18	0.066	0.129
	S-IHI_rest_	− 0.05	− 0.21	0.11	0.548	0.015
	AGE × S-IHI_rest_	0.14	− 0.12	0.41	0.280	0.046
Mirror activity	Intercept	29.51	22.25	36.77	< 0.001	
	AGE	− 25.39	− 38.57	− 12.20	< 0.001	0.408
	S-IHI_active_	− 0.08	− 0.21	0.06	0.251	0.057
	AGE × S-IHI_active_	0.38	0.15	0.62	0.003	0.325
Mirror activity	Intercept	23.15	8.05	38.26	0.004	
	AGE	− 5.86	− 25.38	13.65	0.542	0.015
	L-IHI_rest_	0.07	− 0.15	0.29	0.499	0.018
	AGE × L-IHI_rest_	− 0.03	− 0.31	0.24	0.813	0.002
Mirror activity	Intercept	25.27	12.45	38.08	< 0.001	
	AGE	− 10.13	− 31.30	11.04	0.332	0.041
	L-IHI_active_	0.02	− 0.19	0.22	0.872	0.001
	AGE × L-IHI_active_	0.07	− 0.28	0.42	0.685	0.007

Linear model analysis was conducted with the older group as the reference category. For each model, the interaction was excluded from the model to see a main effect of IHI in case the interaction was not significant (please see the main text)

### Anti-phase tapping task

**Fig. 4:**
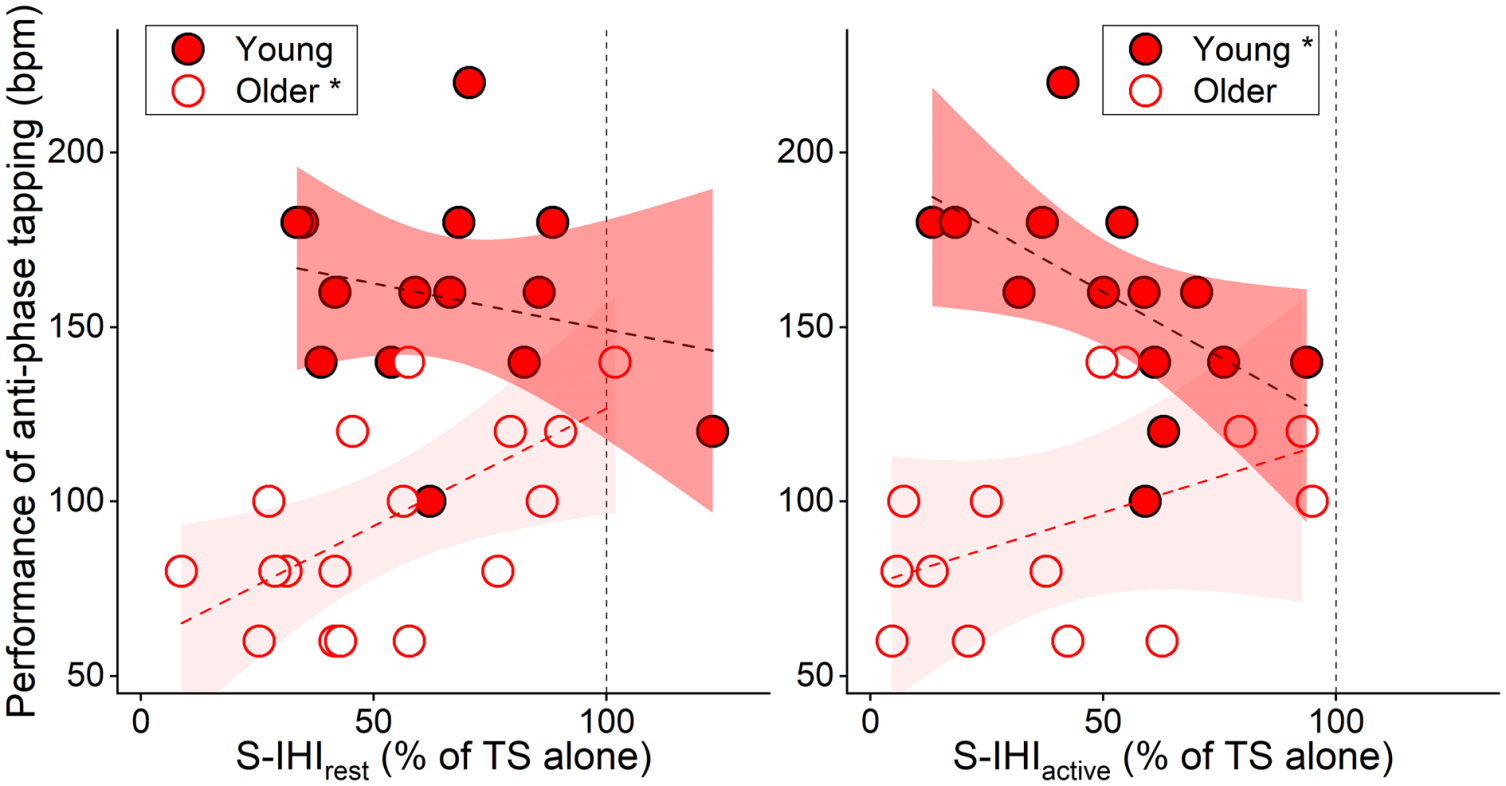
Anti-phase tapping and S-IHI (with 95% confidence interval). *Left*: The analysis demonstrated a significant AGE × S-IHI_rest_ interaction (*p* = 0.036, *η*^2^_*p*_ = 0.152, Table 4), indicating that S-IHI_rest_ influences bimanual performance (dependent variable) differently in the young and older groups: a significant main effect of S-IHI_rest_ in the older group (*p* = 0.026, *η*^2^_*p*_ = 0.170) but not in the young group (*p* = 0.382, *η*^2^_*p*_ = 0.028). *Right*: The analysis also demonstrated a significant AGE × S-IHI_active_ interaction (*p* = 0.008, *η*^2^_*p*_ = 0.258, Table 4): a significant main effect of S-IHI_active_ in the young group (*p* = 0.034, *η*^2^_*p*_ = 0.174) but not in the older group (*p* = 0.080, *η*^2^_*p*_ = 0.122) (dependent variable: bimanual performance). The *y*-axis indicates the performance of anti-phase tapping (beat per minute: bpm) as bimanual performance and the *x*-axis indicates the amplitude of the conditioned MEP amplitude expressed as a percentage of the mean test MEP amplitude evoked by the TS alone showing the degree of S-IHI ([CS + TS]/TS alone × 100) (left, S-IHI_rest_; right, S-IHI_active_). The vertical dashed lines indicate the MEP amplitude evoked by the TS alone (100%). Values below 100% indicate inhibition and values above 100% indicate facilitation. The statistical analysis was conducted with linear model including AGE, S-IHI, and AGE × S-IHI interaction terms, and then separately in the young and older groups since the AGE × S-IHI interactions were significant (Table 4). *N* = 14 in the young due to an incomplete recording (1 young); *N* = 17 in the older (left) due to missing data (5 older without performance of anti-phase tapping); *N* = 14 in the older (right) due to missing data (5 older without performance of anti-phase tapping and 3 older without S-IHI_active_, no overlap). *p < 0.05

The performance of the anti-phase tapping task showed a significant difference between the two groups (two-tailed unpaired *t *test, young vs. older, Anti-phase tapping: 158.6 ± 29.8 vs. 92.9 ± 27.3, *t* = − 6.329, d*f* = 26.8, *p* < 0.001). Linear model analysis for the bimanual performance revealed a significant AGE × S-IHI_rest_ interaction (*p* = 0.036, *η*^2^_*p*_ = 0.152, Fig. 4 and Table 4) with a positive association of S-IHI_rest_ and the bimanual performance in the older group (*p* = 0.026, *η*^2^_*p*_ = 0.170) but not in the young group (*p* = 0.382, *η*^2^_*p*_ = 0.028). In other words, the weaker short-latency inhibitory interaction during the resting state (i.e., S-IHI_rest_), the better the bimanual performance older participants exhibited. Another linear model analysis revealed a significant AGE × S-IHI_active_ interaction (*p* = 0.008, *η*^2^_*p*_ = 0.258, Fig. 4 and Table 4) with a negative association of S-IHI_active_ and the bimanual performance in the young group (*p* = 0.034, *η*^2^_*p*_ = 0.174) but not in the older group (*p* = 0.080, *η*^2^_*p*_ = 0.122). In other words, the stronger short-latency inhibitory interaction from the active to the resting primary motor cortex (i.e., S-IHI_active_), the better the bimanual performance young participants exhibited. L-IHI_rest_ and L-IHI_active_ did neither reveal significant interactions with AGE, nor did they show significant contributions to the bimanual performance after excluding their interaction terms (L-IHI_rest_: *p* = 0.312, *η*^2^_*p*_ = 0.037; L-IHI_active_: *p* = 0.573, *η*^2^_*p*_ = 0.013).

**Table 4:** Linear model results of anti-phase tapping

Outcome	Predictor	Estimate	95% confidence interval		*p* value	*η*^2^_*p*_
			Lower	Upper		
Anti-phase tapping	Intercept	61.42	30.94	91.89	< 0.001	
	AGE	114.30	62.24	166.35	< 0.001	0.429
	S-IHI_rest_	0.60	0.08	1.12	0.026	0.170
	AGE × S-IHI_rest_	− 0.86	− 1.66	− 0.06	0.036	0.152
Anti-phase tapping	Intercept	74.96	50.09	99.83	< 0.001	
	AGE	122.25	76.54	167.96	< 0.001	0.559
	S-IHI_active_	0.42	− 0.05	0.90	0.080	0.122
	AGE × S-IHI_active_	− 1.17	− 2.00	-0.33	0.008	0.258
Anti-phase tapping	Intercept	60.55	19.70	101.41	0.005	
	AGE	99.63	44.71	154.55	< 0.001	0.339
	L-IHI_rest_	0.49	− 0.09	1.07	0.095	0.100
	AGE × L-IHI_rest_	− 0.51	− 1.28	0.25	0.179	0.066
Anti-phase tapping	Intercept	65.63	28.88	102.39	0.001	
	AGE	124.12	61.05	187.20	< 0.001	0.407
	L-IHI_active_	0.49	− 0.11	1.10	0.105	0.106
	AGE × L-IHI_active_	− 1.03	-2.07	0.01	0.051	0.149

Linear model analysis was conducted with the older group as the reference category. For each model, the interaction was excluded from the model to see a main effect of IHI in case the interaction was not significant (please see the main text)

## Discussion

In the present study, IHI by means of dual-site paired-pulse TMS and its role for bimanual movement control were evaluated in healthy young and older adults. In contrast to our hypothesis, no age-related differences of IHI values were observed. In agreement with the previous studies, the amount of mirror activity (Cincotta et al. 2006; Hinder et al. 2011, 2013) and the performance of bimanual asymmetric movement (Fling and Seidler 2012; Serbruyns et al. 2015; Fujiyama et al. 2016a) in the young and older group were different. The main findings of the present study are: (1) S-IHI_active_ demonstrates behavioral relevance for bimanual movement control in young adults, indicating that S-IHI_active_ represents a neurophysiological marker for suppressing activity of the contralateral side; (2) S-IHI_active_ demonstrates different behavioral relevance in young and older adults, suggesting possible age-related functional changes of S-IHI as an adaptive process to the age-related functional decline.

### Changes in IHI from active to resting primary motor cortex during unilateral contraction

IHI may be mediated by interhemispheric excitatory pathways via the corpus callosum and synapse on local inhibitory neural circuits within the target primary motor cortex (Kukaswadia et al. 2005; Lee et al. 2007). Two types of IHI are known with paired-pulse TMS: S-IHI and L-IHI (Ni et al. 2009). Even though evidence suggests that both S-IHI and L-IHI represent transcallosal inhibition (Li et al. 2013), results of previous studies linked two distinct neural circuits to these effects. A pharmacological study reported that L-IHI probably involved GABA_B_ receptors; in contrast, the receptor mediating S-IHI has been still inconclusive (Irlbacher et al. 2007). Furthermore, evidence from patients with callosal infarction suggested that S-IHI and L-IHI may involve different callosal fibers (Li et al. 2013); thus, S-IHI and L-IHI may target different neurons in the contralateral primary motor cortex (Daskalakis et al. 2002; Udupa et al. 2010). The amounts of ipsilateral silent period (iSP) (Chen et al. 2003) and long-interval intracortical inhibition (Kukaswadia et al. 2005; Udupa et al. 2010) showed associations with L-IHI, but not with S-IHI, suggesting common neural circuits between L-IHI and them. Taken together, different underlying circuits and respective behavioral functional relevance between S-IHI and L-IHI are expected.

Previous studies have investigated the effects of unilateral muscle contractions on the IHI from the active to the resting primary motor cortex (i.e., IHI_active_). It has been shown that S-IHI_active_ increases during unilateral muscle contractions compared with S-IHI_rest_ (Ferbert et al. 1992; Perez and Cohen 2008; Vercauteren et al. 2008; Hinder et al. 2010; Morishita et al. 2012; Uehara et al. 2014); however, decreased S-IHI_active_ has been also reported (Perez and Cohen 2008; Nelson et al. 2009; Howatson et al. 2011; Sattler et al. 2012; Turco et al. 2019). In case of L-IHI_active_, no apparent modulation (Sattler et al. 2012; Morishita et al. 2014; Uehara et al. 2014; Turco et al. 2019) and decreased L-IHI_active_ (Nelson et al. 2009) have been reported. These discrepancies could be, at least partly, attributable to methodological differences among previous studies such as different target muscles (distal or proximal), different level of muscle contractions (weak or strong), and adjustment of CS intensity (with or without adjusting CS intensity during unilateral muscle contractions). The discrepancies might be also derived from known variability of MEPs (Kiers et al. 1993; Darling et al. 2006) and therefore related to low test–retest reliability suggested (De Gennaro et al. 2003). For example, a recent study using concurrent TMS-EEG showed high test–retest reliability of interhemispheric dynamics (Casula et al. 2020), which might be crucial considering the discrepancies. In addition to the above, we argue that increased or decreased IHI_active_ could simply have been due to the neurophysiological characteristics of each individual linked to behavior (details below).

### Contribution of IHI to bimanual movement control

Although previous studies have shown the general importance of the corpus callosum for bimanual movement control (Eliassen et al. 2000; Kennerley et al. 2002), more detailed evidence of the role of inhibitory and facilitatory interhemispheric interactions between primary motor cortices in healthy adults is missing. Here, we demonstrated that S-IHI showed a behavioral impact on bimanual movement control. Notably, the greater degree of S-IHI_active_ was associated with the less amount of mirror activity (Fig. 3) and the better performance of anti-phase tapping (Fig. 4) in young adults. Mirror activity is associated with additional motor cortical activity (Tsuboi et al. 2010; Soteropoulos et al. 2011); thus, the results suggest that S-IHI_active_ represents a neurophysiological marker for suppressing activity of the contralateral side when the primary motor cortex ipsilateral to the active hand is active. However, it is important to emphasize that bimanual asymmetric movements involve not solely interhemispheric interactions between the primary motor cortices, but also within secondary motor areas especially the dorsal premotor cortex (Meyer-Lindenberg et al. 2002; Liuzzi et al. 2011; Verstraelen et al. 2021) as well as prefrontal areas. In case of the dorsal premotor cortex and prefrontal areas, the interactions from the left to the right hemisphere might mainly contribute to bimanual movement control (Fujiyama et al. 2016a, b). Thus, the present data add further evidence that both inhibitory (primary motor cortex, shown in the present study) and facilitatory (secondary motor and prefrontal areas, shown in previous studies, e.g., Liuzzi et al. 2011; Fujiyama et al. 2016a) interhemispheric interactions are crucial for successful bimanual movement control. The stronger short-latency inhibitory interaction from the active to the resting primary motor cortex, the more capacity to suppress activity of the contralateral side likely contributing to efficient bimanual movement control. Behavioral relevance of S-IHI_active_ suggests that a comparison between IHI_rest_ and IHI_active_ would be confounded by an individual’s ability to perform bimanual movements. This could explain, at least partly, incoherent findings in the previous studies examining IHI_active_ without considering its behavioral relevance.

Our analyses demonstrated that L-IHI_active_ did not contribute to the amount of mirror activity nor the performance of anti-phase tapping. The present results add to the notion that S-IHI and L-IHI represent two distinct neural circuits by showing different behavioral relevance for bimanual movement control. L-IHI_active_ may be differently involved in interhemispheric interactions with a subsequent functional role compared with S-IHI_active_, which needs to be addressed in future investigations.

### Possible age-related functional changes of IHI

It should be noted that the behavioral associations of S-IHI_active_ were only found in the young group. The present results showed no apparent difference of IHI between the two groups—at least partly in line with the previous studies for S-IHI (Hinder et al. 2010; Hermans et al. 2018) but for L-IHI (Mooney et al. 2018)—despite the behavioral differences. The first possible explanation for the lack of behavioral associations in the older group could be that the amount of mirror activity and the performance of anti-phase tapping in older adults are rather associated with neural circuits other than the one represented by S-IHI_active_. That is, other neural mechanisms might possess a larger contribution for directing bimanual movement control in older adults such as prefrontal areas (Fujiyama et al. 2016a), intracortical inhibition (Mooney et al. 2017; Hermans et al. 2018), or GABA levels (Cuypers et al. 2020). Another possible explanation might be an insufficient degree of S-IHI_active_ suppressing activity of the contralateral side. Older adults have been shown to recruit wider areas of the brain, such as bilateral motor cortical areas, even during simple unilateral tasks (e.g., Ward et al. 2008). Thus, it might be the case that the degree of S-IHI_active_ in the older group was insufficient to suppress activity of the contralateral side, which could be shown as the lack of behavioral associations.

An alternative explanation for the lack of behavioral associations in the older group could be associated with a possible age-related functional change of IHI. The present data suggest that the IHI values in the older group are associated between the two states (i.e., IHI_rest_ and IHI_active_) (Fig. 2). From a different point of view, IHI values in the young group demonstrate rather distinct patterns (Fig. 2). This may suggest that each IHI value could represent different functional meaning; this could also support the view that IHI_rest_ and IHI_active_ represent different neurophysiological markers with different functional relevance. A lack of distinct IHI values in the older group might reflect a loss of functional capacity of IHI, especially S-IHI_active_ suppressing activity of the contralateral side. We propose that this loss of functional capacity of S-IHI_active_ might derive from an age-related adaptive change of IHI. In the older group, the less degree of S-IHI_rest_ was associated with the better performance of anti-phase tapping (Fig. 4, left), as reported previously (Fling and Seidler 2012). Based on the assumed function of IHI, this is counterintuitive and not entirely clear why such a behavioral association is present in older adults. Although it is speculation at this point, one possible explanation for the result of S-IHI_rest_ might be related to bilateral cortical activity seen in older adults (Ward et al. 2008); these age-related changes are generally anticipated as adaptive processes, likely supporting the best processing of motor tasks (Mattay et al. 2002; Heuninckx et al. 2008; Zimerman et al. 2014). Although bilateral cortical activity is not only present in the primary motor cortex but also wide areas of the brain, less S-IHI_rest_ could represent one possible mechanism to recruit wider bilateral areas of the brain as an age-related adaptive change, resulting in better performance and/or avoiding worse performance. This adaptive change of IHI might result in, as a consequence, a loss of functional capacity of S-IHI_active_ suppressing activity of the contralateral side. An age-related functional change of IHI might be one possible adaptive process to compensate behavioral decline during aging.

### Methodological consideration

A few previous studies reported, at first glance, inconsistent findings with the present results. Notably, the inconsistencies rather point out crucial methodological and neurophysiological considerations; therefore, we discuss about them in more detail in the following section. In spite of a different way to assess bimanual performance, a previous study reported a contradicting result: less transcallosal inhibition was associated with better bimanual performance in young adults (Fling and Seidler 2012). In their study, transcallosal inhibition was assessed with iSP, inducing inhibition in the direction resting to active primary motor cortex. However, the evaluated performance of the bimanual movement addressed primarily the effects from the active primary motor cortex (i.e., the opposite directionality to the assessed iSP). Giovannelli et al. (2009) reported results similar to our findings: the greater iSP was associated with the less amount of mirror activity in young adults (Giovannelli et al. 2009). In their study, iSP was assessed quantifying inhibition in the direction from the active primary motor cortex (i.e., the same directionality to the assessed mirror activity).

Hübers et al. (2008) reported that the degree of S-IHI_rest_ was associated with the amount of mirror activity. However, this association was not found by Bologna et al. (2012), nor in the present study (Table 3). The inconsistencies could partly derive from differences in the assessment of mirror activity and the statistical approach used to examine associations of S-IHI_rest_ with the amount of mirror activity. For instance, Hübers et al. (2008) examined 13 young adults and data from both hands were simply combined in a regression model without considering the dominant or non-dominant hands. Furthermore, they evaluated multiple IHI values with different CS intensities. Previous studies have shown that S-IHI_rest_ and S-IHI_active_ from the dominant to non-dominant primary motor cortex and from the non-dominant to dominant primary motor cortex may significantly differ (Netz et al. 1995; Bäumer et al. 2007). Thus, the inconsistency could also derive from the potential asymmetry of S-IHI and its association with mirror activity. According to a recent study showing functional relevance of the symmetry of interhemispheric propagation for stroke patients (Casula et al. 2021), the potential symmetry/asymmetry of IHI and its association with bimanual movement control have to be addressed in more detail in upcoming studies. Nevertheless, the degree of S-IHI_rest_ could still represent behavioral relevance for bimanual movement control (Hübers et al. 2008; Wahl et al. 2016).

One could argue that the assessment of IHI_active_ in the present study was not performed during the assessments of bimanual movement. The rationale for the present approach was that if IHI_active_ was assessed during the assessments of bimanual movement, it would be problematic to interpret whether the amount of IHI_active_ leads to bimanual performance or whether bimanual performance leads to the amount of IHI_active_. Due to this, we decided to apply an associative approach by correlating the degree of IHI with the performance of bimanual tasks. Moreover, Hinder et al. (2010) examined the degree of IHI_active_ during isometric and phasic contractions, and showed that the degrees of IHI_active_ in two contraction modes did not differ (Hinder et al. 2010). Thus, we propose that IHI_active_ represents the general inhibitory capacity from the active to the resting primary motor cortex during voluntary movements.

### Functional implication

Despite the notion that TMS probes only a subgroup of descending motor fibers and artificial activation of descending connections (for review, Bestmann and Krakauer 2015), the present results demonstrate that IHI exhibits behavioral relevance for bimanual movement control. Notably, the results suggest that S-IHI_active_ represents a neurophysiological marker for suppressing activity of the contralateral side, likely contributing to efficient bimanual movement control. Our analyses indicate that behavioral relevance of IHI for bimanual movement control differs in older adults, suggesting an age-related functional change of IHI. Weaker inhibitory interactions between the two hemispheres (i.e., less IHI) might be one possible mechanism of adaptive processes to compensate behavioral decline during aging. Given the demographic development in modern societies, understanding and preventing this decline, even supporting adaptive processes, are of outmost importance. For example, it has been suggested that physical exercises and non-invasive brain stimulation of prefrontal cortex could modulate inhibitory interactions (Verstraelen et al. 2020; Levin et al. 2021), which might improve not only bimanual movement control but also inhibitory control in general in older adults.

IHI has been investigated in many neurological disorders (e.g., Murase et al. 2004; Duque et al. 2005; Li et al. 2007; Beck et al. 2009) and such investigations have provided valuable insights into the pathophysiological understanding. As many neurological disorders occur at a later stage of life, the results of the present study will add to the better understanding of disease-related changes of interhemispheric interactions and their behavioral impact, especially disentangling age- and pathology-related changes of IHI.

## Data Availability

The data generated during and/or analyzed during the current study are available from the corresponding author on reasonable request.
